# Integrative taxonomy of *Xiphinema histriae* and *Xiphinema lapidosum* from Spain

**DOI:** 10.21307/jofnem-2019-037

**Published:** 2019-07-29

**Authors:** Ruihang Cai, Antonio Archidona-Yuste, Carolina Cantalapiedra-Navarrete, Juan E. Palomares-Rius, Jingwu Zheng, Pablo Castillo

**Affiliations:** 1 Institute for Sustainable Agriculture (IAS), Spanish National Research Council (CSIC), Campus de Excelencia Internacional Agroalimentario, ceiA3, Avenida Menéndez Pidal s/n, 14004, Córdoba, Spain; 2 Laboratory of Plant Nematology, Institute of Biotechnology, College of Agriculture and Biotechnology, Zhejiang University, Hangzhou, 310058, Zhejiang, P.R. China.

**Keywords:** First record, Juvenile stages, Molecular, Morphology, Morphometrics, Phylogeny, *Xiphinema*, *X. histriae*, *X. lapidosum*

## Abstract

Three populations of *Xiphinema* non-*americanum* group species were detected in agricultural and natural ecosystems, during routine surveys for plant-parasitic nematodes in Spain. Based on morphological and molecular analyses, the species were identified as *Xiphinema histriae* and *Xiphinema lapidosum*, being this the first record and molecular characterization of both species in Spain. The morphometrics and morphology of the Spanish populations agree with those of the original description and paratype specimens and the present study provided a first description of the second to fourth juvenile stages of both species. A detailed study on the morphology in the Spanish populations of *X. histriae*, as well as in paratypes, showed a pseudo-Z-organ with weakly muscularized wall and containing numerous small dense granular bodies, which was different to the original description by Lamberti et al. (1993). This new finding suggests that *X. histriae* must be considered a member of the morphospecies Group 5 of *X.* non-*americanum*. Phylogenetic analysis based on D2 to D3 expansion segments of 28S gene, ITS1 and partial CoxI gene indicated that *X. histriae* and *X. lapidosum* are phylogenetically related with other *Xiphinema* non-*americanum* group spp. reported from Spain. Considering the pathological and economic importance of this group of nematodes, the combination of morphological characters, measurements, and molecular analysis is crucial for accurate identification of these species.

The genus *Xiphinema* Cobb, 1913 is a large and morphologically diverse group of plant-parasitic nematodes comprising more than 275 species (Archidona-Yuste et al., 2016a, 2016b; Peraza-Padilla et al., 2018). The economic importance of this group of nematodes is not only because of its extensive range of host plants and worldwide distribution, but for the transmission of several important plant viruses (genus *Nepovirus*, family Comoviridae) that cause direct damage to a wide variety of crops (Taylor and Brown, 1997; Decraemer and Robbins, 2007). Due to their economic importance, complex identification because of the sharing of a variety or morphological characters and existence of cryptic species, it is essential to identify species accurately and developing integrative taxonomy methods to control such plant pathogenic species (Archidona-Yuste et al., 2016a, 2016b). Species identification in this group is complex because of the sharing of a variety of morphological characters and the existence of cryptic species (Archidona-Yuste et al., 2016a, 2016b). According to the key for species of *Xiphinema* established by Loof and Luc (1990), the genus *Xiphinema* consists of *X. americanum*-group and *X*. non-*americanum* species. Later, non-*americanum* group was divided into eight morphospecies groups (Loof and 1990). Several authors have highlighted the great diversity of *Xiphinema* spp. detected in the Iberian Peninsula, in particular, around 40 species of the genus *Xiphinema* have been reported in Spain, mainly associated with woody, ornamental, and vegetable plant species (Gutiérrez-Gutiérrez et al., 2010, 2013, 2016; Archidona-Yuste et al., 2016a, 2016b).

Routine nematological surveys in agricultural and natural ecosystems in Spain yielded three populations of *Xiphinema* non-*americanum* group species, which were typologically different to previous reported species in Spain. Two populations of *Xiphinema histriae* were isolated from *Quercus faginea* Lam. and *Pinus nigra* Arnold, whereas one population of *Xiphinema lapidosum* was identified in association with *Olea europaea* subsp. *europaea* L. Lamberti et al. (1993a, 1993b) and Roca and Bravo (1993) described female and male stages of *X. histriae* and *X. lapidosum*, respectively, but in both species no juvenile stages were detected and described. The objectives of this study were: (i) to provide updated morphological descriptions of juvenile stages of *X. histriae* and *X. lapidosum*, (ii) to characterize the molecular data of both species using the D2 to D3 segments, ITS1 and partial CoxI gene sequences, and (iii) to determine the phylogenetic relationships of both species within the *X*. non-*americanum* group species.

## Materials and methods

### Nematode sampling, extraction, and morphological study

Nematodes were surveyed from 2017 to 2018 during the spring season in natural ecosystems and olive growing area in Andalucia, southern Spain (Table 1). Soil samples were collected for nematode analysis with a shovel from four to five cores randomly selected in each sampling site. Nematodes were extracted from a 500-cm^3^ sub-sample of soil by a modification of Cobb’s decanting and sieving method (Flegg, 1967). Specimens were killed and fixed with hot formalin (4% with 1% glycerol), and processed in glycerin (Seinhorst, 1959) as modified by De Grisse (1969). The measurements and light micrographs of nematodes were performed using a Zeiss III compound microscope.

**Table 1.** Taxa sampled for *Xiphinema* species and sequences from NCBI used in this study.

Species	Sampling code	Locality	Host-plant	D2 to D3	ITS1	CoxI
**1.** ***X. histriae***	**QUECAZ** ^**a**^	**Arroyo Frio, Cazorla, Cazorla, Jaén province, Spain**	**Portuguese oak**	**MK801302**	**MK801298**	**MK796911**
				**MK801303**		
	**NAVSAP**	**Navas de San Pedro, Cazorla, Jaén province, Spain**	**Black pine**	**MK801304**	**MK801299**	**MK796912**
				**MK801305**		
**2.** ***X. lapidosum***	**JAO132**	**Aroche, Huelva province, Spain**	**Cultivated olive**	**MK801306**	**MK801300**	**MK796913**
				**MK801307**		**MK796914**
						**MK796915**
3. *X. andalusiense*	419b	Andujar, Jaén province, Spain	Wild olive	KX244886	–	–
	AR108	Villaviciosa, Córdoba province, Spain	Wild olive	–	–	KY816595
4. *X. abrantinum*	CAN223	Portugal	–	AY601625	–	–
5. *X. aceri*	M13	Maragheh city, Iran	Wild rose	–	EU477385	–
6. *X. adenohystherum*	–^b^	Bollullos par del Condado, Huelva province, Spain	Grapevine	GU725075	GU725063	–
	AR086	Prado del Rey, Cádiz province, Spain	Wild olive	–	–	KY816590
7. *X. baetica*	H001	Hinojos, Huelva province, Spain	Stone pine	KC567169	–	–
	LOMAS	Hinojos, Huelva province, Spain	Stone pine	–	–	KY816596
8. *X. bakeri*	CD947	Olympic Peninsula, Washington, USA	Unknown	KF292276	–	–
	CD852	Point Reyes, Marin county, California, USA	Unknown	–	–	KF292305
9. *X. barense*	CNR1	Brindisi province, Italy	Wild olive	KM199691	–	–
	its1IAS	Brindisi province, Italy	Wild olive	–	KM199693	–
	itsLUC	Brindisi province, Italy	Wild olive	–	KM199694	–
10. *X. basiri*	EU126	San Jose, Cuba	–	AY601630	–	–
11. *X. belmontense*	MOUB	Merza, Coruna, Spain	Pedunculate oak	KC567172	–	–
	MOUCH	Merza, Pontevedra province, Spain	Chestnut	–	–	KY816598
12. *X. brasiliense*	SZX1305	Shenzhen, Guangdong province, China	Resam	KP793050	–	–
13. *X. cadavalense*	ST77	Espiel, Córdoba province, Spain	Cultivated olive	KX244900	–	KY816599
14. *X. castilloi*	–	Gilan province, Iran	Ash	KF446655	–	–
15. *X. celtiense*	AR83	Penaflor, Sevilla province, Spain	Wild olive	KX244889	KX244926	–
	AR82	Adamuz, Córdoba province, Spain	Wild olive	–	KX244927	KY816601
16. *X. chambersi*	1,602	Jekyll Island, Georgia, USA	Oak	KU680967	–	–
	3,357	Jekyll Island, Georgia, USA	Oak	–	–	KU764419
17. *X. cohni*	AR16	Sanlucar de Barrameda, Cádiz province, Spain	Wild olive	KX244901	KX244933	–
	J126-2	El Puerto de Santa Maria, Cádiz, Spain	Stone pine	–	KC567159	–
	J0126	El Puerto de Santa Maria, Cádiz, Spain	Grapevine	–	–	KY816602
18. *X. conurum*	ST45	Uleila del Campo, Almería province, Spain	Cultivated olive	KX244902	–	–
	ST45V	Sorbas, Almería province, Spain	Cultivated olive	–	–	KY816603
19. *X. costaricense*	ACC61	La Suiza de Turrialba, Cartago province, Costa Rica	Sugarcane	KX931059	–	–
	ACC46	Santa Rosa, Limon province, Costa Rica	Cocoa	–	–	KY816605
20. *X. coxi*	GG10	Glynn County, Georgia, USA	–	AY601631	–	–
21. *X. coxi europaeum*	AR92	Alcolea, Córdoba province, Spain	Wild olive	KX244903	–	–
	AR020	Hinojos, Huelva province, Spain	Wild olive	–	–	KY816606
22. *X. cretense*	OLI40	Hersonisos province, Greece	Olive	KJ802879	KJ802895	–
	AR039	Hersonisos province, Greece	Wild olive	–	–	KY816608
23. *X. dentatum*	–	Silnicna, Czech Republic	Hornbeam, Norway maple	EU781538	–	EU781537
24. *X. diversicaudatum*	KOS	Klucovec, Slovakia	Unknown	JQ780367	–	–
	AUS	Marchegg, Austria	Unknown	–	–	GU222423
25. *X. elongatum*	CD426	Brisbane, Australia	Grasses	MF510431	–	MF510426
26. *X. gersoni*	H0059	Almonte, Huelva province, Spain	Eucalyptus	KC567180	–	KY816610
27. *X. globosum*	–	Alcalá de los Gazules, Cádiz Province, southern Spain	Unknown	GU549474	–	–
28. *X. granatum*	Xmar1	Markazi province, Iran	Pomegranate	JQ240273	–	–
29. *X. hangzhouense*	–	Hangzhou, Zhejiang province, China	Bull Bay	MF538772	–	MF706262
30. *X. herakliense*	OLE18	Agiofarago, Crete Island, Greece	Wild olive	KM586349	–	KY816613
	OLE17	Agiofarago, Crete Island, Greece	Wild olive	–	KM586355	–
	OLE16	Agiofarago, Crete Island, Greece	Wild olive	–	KM586354	–
31. *X. hispanum*	–	Andújar, Jaén province, Spain	Estepa blanca	GU725074	GU725061	–
	00419	Andújar, Jaén province, Spain	Wild olive	–	–	KY816614
32. *X. hispidum*	AR004	Medina Sidonia, Cádiz province, Spain	Wild olive	KX244906	–	–
	Xhi426	Bollullos par del Condado, Huelva province, Spain	Grapevine	–	HM921367	–
	H026b	Rociana par del Condado, Huelva province, Spain	Grapevine	–	–	KY816616
33. *X. hunaniense*	CD2465	Thailand	–	MF510432	–	MF510427
34. *X. ifacolum*	AE90	Kalutara province, Sri Lanka	Grasses	MH012181	–	MH013396
35. *X. index*	XinTre	Trexenta, Cagliari province, Italy	Grapevine	HM921406	–	HM921388
36. *X. ingens*	Ps	Kermanshah province, Iran	Unknown	KJ956388	–	–
37. *X. insigne*	CD1238	Fresno County, California, USA	Grasses	MF510430	–	MF510425
38. *X. iranicum*	M46	Maragheh city, Iran	wild rose	–	EU477386	–
39. *X. israeliae*	OLI34	Voutes province, Greece	Olive	KJ802886	–	–
	OLI13	Roufas province, Greece	Olive	–	KJ802896	–
	AR013	Roufas province, Greece	Wild olive	–	–	KY816618
40. *X. italiae*	AR91	Puerto Real, Cádiz province, Spain	Wild olive	KX244912	KX244937	–
	XIP12	Sbiba, Kasserine province, Tunisia	Cultivated olive	–	KX062698	–
	APUL	Bari, Bari province, Italy	Grapevine	–	–	KY816623
41. *X. iznajarense*	JAO25	Iznajar, Córdoba province, Spain	Cultivated olive	KX244892	KX244928	KY816624
					KX244929	
42. *X. japonicum*	JH-2017	Japan	Arhat pine	–	–	KY628214
43. *X. krugi*	CD1827	Matapalo, Puntaneras province, Costa Rica	Rubber Plant	KX931063	–	–
	ACC13	Santa Gertrudis, Alajuela province, Costa Rica	Sugarcane	–	–	KY816626
44. *X. lupini*	H050	Hinojos, Huelva, Spain	Grapevine	KC567183	–	–
	388GD	Bollullos par del Condado, Huelva, Spain	Grapevine	–	–	KY816630
45. *X. macroacanthum*	individual 70	Adriatic Sea coast, Italy	Olive	HF546081	–	–
	ITAL	Bridisi province, Italy	Cultivated olive	–	–	KY816631
46. *X. macrodora*	JAO6	La Granjuela, Córdoba province, Spain	Cultivated olive	KU171041	–	–
	AR097	Santa Mª de Trassierra, Córdoba province, Spain	Wild olive	–	–	KY816632
47. *X. meridianum*	XMP11	Sbitla, Kasserine province, Tunisia	Cultivated olive	KX062679	–	–
	11R16	Sbitla, Kasserine province, Tunisia	Common buckthorn	–	–	KY816635
48. *X. mengibarense*	OC3C4	Mengibar, Jaén province, Spain	Cultivated olive	KX244895	–	–
	O3V05	Mengibar, Jaén province, Spain	Cultivated olive	–	–	KY816634
49. *X. naturale*	N1	Fort Pierce, Florida, USA	Oak	DQ299515	–	–
50. *X. nuragicum*	JAO36	Casarabonela, Malaga province, Spain	Wild olive	KX244913	–	–
	RONDA	Ronda, Malaga province, Spain	Abete di Spagna	–	GU725059	–
	AR113	Alcolea, Córdoba province, Spain	Wild olive	–	–	KY816640
51. *X. oleae*	AR35	Tarifa, Cádiz province, Spain	Wild olive	KU171038	–	–
52. *X. parachambersi*	–	Japan	Cape jasmine, Spindle	MG786445	–	–
53. *X. paradentatum*	17-82	Tara Mountain, Serbia	Meadow	LT883658	–	–
54. *X. poasense*	AJ74	San Carlos, Alajuela province, Costa Rica	Eucalyptus, Cypress and Fountain grass	MF461347	–	–
	AP99	San Carlos, Alajuela province, Costa Rica		–	–	MF461335
55. *X. pseudocoxi*	AR95	Alcaracejos, Córdoba province, Spain	Wild olive	KX244915	–	KY816643
56. *X. pyrenaicum*	PYRDJ	Cahors, Midi-Pyrenees, France	Grapevine	GU725073	GU725060	–
57. *X. radicicola*	V1273	Chu’momray, Viet Nam	–	AY601622	–	–
58. *X. robbinsi* Topotype	TypPop	Goshayesh village, East Azarbaijan, Iran	Common buckthorn	MH744579	–	–
59. *X. robbinsi* Tunisia	12R28	Sbitla, Kasserine province, Tunisia	Cultivated olive	KX062683	–	KY816647
60. *X. savanicola*	CAN72	Dakar, Senegal	–	AY601620	–	–
61. *X. setariae*	AC009	Pueblo Nuevo de Duacarí, Limón, Costa Rica	Banana	KX931066	–	KY816648
62. *X. sphaerocephalum*	AR73	Castillo de Locubín, Jaén province, Spain	Wild olive	KX244917	–	–
	AR63	Coto Ríos, Jaén province, Spain	Oak	–	GU725062	–
	AR063	Coto Ríos, Jaén province, Spain	Wild olive	–	–	KY816649
63. *X. tica*	ACC81	Sabanillas, San Jose province, Costa Rica	Coffeae	KY623491	–	–
	ACC32	Lagunilla, Guanacaste province, Costa Rica	Soursop	–	–	KY623501
64. *X. turcicum*	ST149	Prado del Rey, Cádiz province, Spain	Cultivated olive	KX244919	–	–
	ST149	San José del Valle, Cádiz province, Spain	Wild olive	–	–	KY816650
65. *X. turdetanense*	J212	Sanlucar de Barrameda, Cádiz Province, Spain	Stone pine	KC567186	–	–
	AR015	Sanlucar de Barrameda, Cádiz Province, Spain	Wild olive	–	–	KY816651
66. *X. vuittenezi*	–	Slany, Czech Republic	Apple	EF614266	–	EF614265
	–	Rhein Valley, Germany	–	–	AJ437028	–
	population O	Romania	Grapevine	–	HG329722	–
67. *X. zagrosense*	Yasooj	Zagros Mountains, Iran	Grass	JN153101	–	–

Note: ^a^Newly sequences species in bold font; ^b^(–) data not provided or sequence not used.

A comparative morphological and morphometrical study of type specimens of *X. histriae* were conducted with specimens kindly provided by Dr A. Troccoli, from the nematode collection at the Istituto per la Protezione Sostenibile delle Piante (IPSP), Consiglio Nazionale delle Ricerche (CNR), Bari, Italy; and paratypes of *X. lapidosum* kindly provided by Dr Z.A. Handoo from USDA Nematode Collection, Beltsville, MD, USA (T-4406p; T4407p). Spanish nematode populations of both *Xiphinema* species in this study are proposed as standard and reference populations for each species given until topotype material becomes available and molecularly characterized. Voucher specimens of these described species have been deposited in the nematode collection of Institute for Sustainable Agriculture, IAS-CSIC, Córdoba, Spain.

### Molecular analyses

For molecular analyses, in order to avoid mistakes in the case of mixed populations, two live nematodes from each sample were temporary mounted in a drop of 1M NaCl containing glass beads (to avoid nematode crushing/damaging specimens) to ensure specimens conformed to the unidentified populations of *Xiphinema*. Following morphological confirmation, the specimens were removed from the slides and DNA extracted. DNA was extracted from single specimens as described by Archidona-Yuste et al. (2016a, 2016b). The D2 to D3 segments were amplified using the D2A (5’-ACAAGTACCGTGAGGGAAAGTTG-3’) and D3B (5’-TCGGAAGGAACCAGCTACTA-3’) primers (De Ley et al., 1999). The ITS1 region was amplified using forward primer 18S (5’-TTGATTACGTCCCTGCCCTTT-3’) (Vrain et al., 1992) and reverse primer rDNA1 5.8S (5’-ACGAGCCGAGTGATCCACCG-3’) (Cherry et al., 1997). And CoxI gene was amplified as described by Lazarova et al. (2006) using the primers COIF (5’-GATTTTTTGGKCATCCWGARG-3’) and COIR (5’-CWACATAATAAGTATCATG-3’). The newly obtained sequences were submitted to the GenBank database under accession numbers indicated on the phylogenetic trees and in Table 1.

### Phylogenetic analysis

D2 to D3 segments, partial ITS1 rRNA, and partial CoxI sequences of different *Xiphinema* species belonging to the *X.* non-*americanum* group were obtained from GenBank and used for phylogenetic reconstruction. Outgroup taxa for each data set were chosen following previous published studies: *Longidorus oleae* (KT308871), *Xiphinema americanum* (KX263175); *Longidorus caespiticola* (KJ567469), *Xiphinema duriense* (KX244935), *Xiphinema pachtaicum* (HM921337); *Scutellonema bradys* (AY268114), *Meloidogyne hapla* (AY268113) (He et al., 2005; Holterman et al., 2006; Gutiérrez-Gutiérrez et al., 2013; Tzortzakakis et al., 2015; Archidona-Yuste et al., 2016a, 2016b; Susulovska et al. 2018: Varela-Benavides et al., 2018). Multiple sequence alignments of the different genes were made using the Q-INS-i algorithm of MAFFT V.7.205 (Katoh and Standley, 2013), which accounts for secondary RNA structure. Sequence alignments were visualized and their percentage of similarity calculated using the sequences identity matrix using BioEdit (Hall, 1999) and manually edited by Gblocks ver. 0.91b (Castresana, 2000) in Castresana Laboratory server (http://molevol.cmima.csic.es/castresana/Gblocks_server.html) using options for a less stringent selection (minimum number of sequences for a conserved or a flanking position: 50% of the number of sequences  + 1; maximum number of contiguous non-conserved positions: 8; minimum length of a block: 5; allowed gap positions: with half).

Phylogenetic analyses of the sequence data sets were based on Bayesian inference (BI) using MrBayes 3.1.2 (Ronquist and Huelsenbeck, 2003). The best-fit model of DNA evolution was obtained using JModelTest V.2.1.7 (Darriba et al., 2012) with the Akaike Information Criterion (AIC). The best-fit model, the base frequency, the proportion of invariable sites, the gamma distribution shape parameters, and substitution rates in the AIC were then given to MrBayes for the phylogenetic analyses. BI analyses were performed under the general time-reversible model with invariable sites and a gamma-shaped distribution (GTR +  I  +  G) for the D2 to D3 segments of 28S, rRNA ITS1 and partial CoxI gene. These BI analyses were run separately per data set using four chains for 2 × 10^6^ generations for all of molecular markers. The Markov chains were sampled at intervals of 100 generations. Two runs were conducted for each analysis. After discarding burn-in samples and evaluating convergence, the remaining samples were retained for further analyses. The topologies were used to generate a 50% majority rule consensus tree. Posterior probabilities (PP) are given on appropriate clades. Trees from all analyses were visualized using FigTree software V.1.42 (http://tree.bio.ed.ac.uk/software/figtree/).

## Results

Systematics

**Figure 1.**
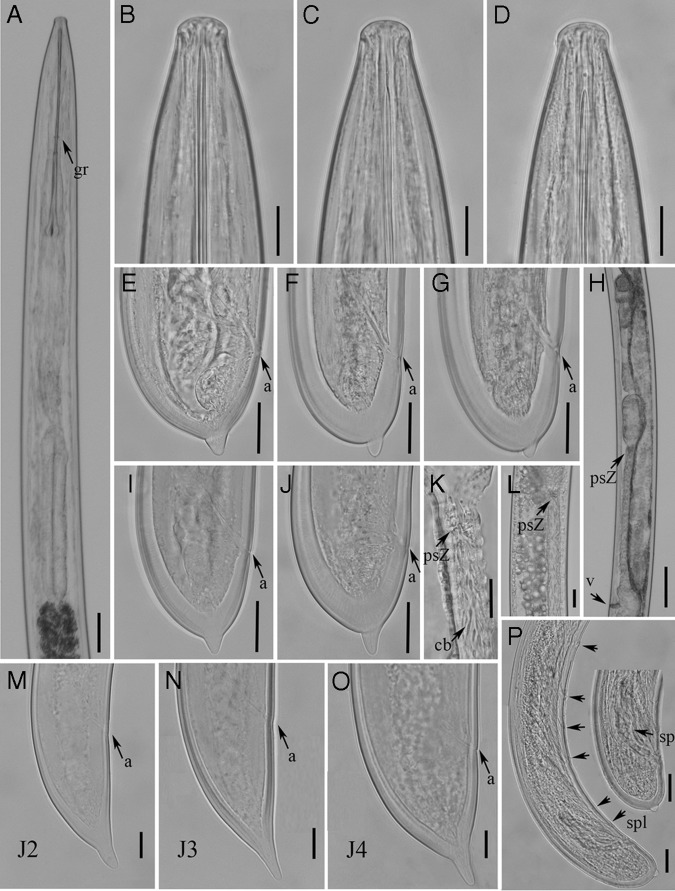
Light micrographs of *Xiphinema histriae* (Lamberti et al., 1993a, 1993b). Females: A, Pharynx; B to D, Lip regions; E to G, I, J, Tail region; H, Gonad; K, L, Details of pseudo-Z-organ; Juveniles: M to O, Tail region of 2nd, 3rd and 4th stage juveniles; Males: P, Tail region of male. Abbreviations: a, anus; cb, crystalloid bodies; gr, guiding ring; psZ, pseudo-Z-organ; sp, spicule; spl, supplements; v, vulva. (Scale bars: A = 40 μm; B-D = 15 μm; E–G, I, J = 20 μm; H = 65 μm; K = 10 μm; L = 20 μm; M–O = 10 μm; P = 20 μm.)

**Figure 2:**
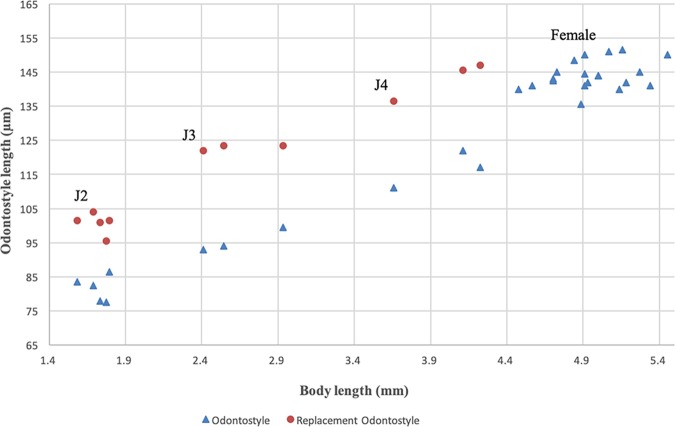
Relationship of body length to length of functional and replacement odontostyle (▴=Odontostyle and •=Replacement odontostyle); length in three developmental stages and mature females of *Xiphinema histriae*.

**Figure 3.**
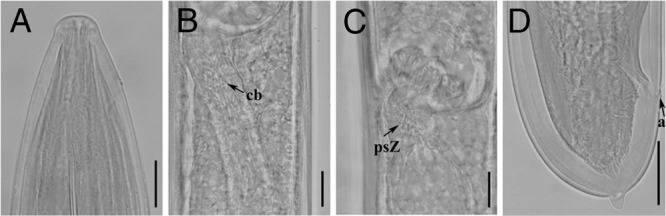
Light micrographs of *Xiphinema histriae* paratypes (Lamberti et al., 1993a, 1993b). Female: A, Lip region; B, C, Details of pseudo-Z-organ; D, Tail region. Abbreviations: a, anus; cb, crystalloid bodies; psZ, pseudo-Z-organ. (Scale bars: A = 15 μm; B, C = 10 μm; D = 20 μm.)

*Xiphinema histriae* Lamberti et al. (1993a, 1993b).

(Figs. 1–3; Table 2).

**Table 2.** Comparative morphometrics of females and males of *Xiphinema histriae* (Lamberti et al., 1993a, 1993b) from different localities. All measurements are in *µ*m and in the form: mean ± sd (range)^a^.

Locality/host-plant	Arroyo Frío, Cazorla Jaén, Spain, Portuguese oak				Nava de San Pedro, Cazorla Jaén, Spain, pine		Paratypes, (Lamberti et al., 1993a) Gorizia, Italy, *Vitis* sp.		(Lamberti et al., 1993b) Trieste, Italy, *Vitis* sp.	
Characters/ratios^b^	Females	J2	J3	J4	Females	Male	Females	Males	Females	Male
n	20	5	3	3	11	1	5	3	4	1
L^b^	4,959.0 ± 253.7 (4,477.0–5,455.0)	1,715.0 ± 83.9 (1,583.0–1,795.0)	2,629.0 ± 271.4 (2,409.0–2,932.0)	4,000.0 ± 300.7 (3,659.0–4,227.0)	4,762.4 ± 322.4 (4,364.0–5,364.0)	4,773.0	4.3 ± 0.19 (4.0–4.5)	4.2 ± 0.21 (4.0–4.3)	4.2 (4.1–4.5)	4.5
a	77.6 ± 5.4 (68.2–86.4)	41.5 ± 4.0 (37.7–46.7)	57.4 ± 2.7 (54.8–60.2)	68.2 ± 1.4 (67.1–69.7)	73.0 ± 3.9 (67.1–79.2)	68.2	67.9 ± 2.2 (64.1–69.4)	65.9 ± 0.8 (65.4–66.5)	68.4 (61.0–78.1)	78.8
b	8.7 ± 0.6 (7.4–10.2)	4.3 ± 0.4 (3.7–4.7)	5.8 ± 0.5 (5.4–6.3)	7.1 ± 0.1 (7.1–7.3)	8.9 ± 0.8 (8.1–10.6)	7.6	8.3 ± 0.5 (7.7–9.0)	8.0 ± 0.4 (7.7–8.3)	8.1 (7.3–9.3)	9.1
c	111.4 ± 8.4 (98.4–130.2)	32.6 ± 1.5 (31.0–34.5)	45.3 ± 4.4 (40.4–48.9)	73.8 ± 3.4 (71.0–77.6)	114.1 ± 7.7 (99.7–128.1)	116.4	103.7 ± 8.5 (96.6–117.8)	82.5 ± 4.9 (79.0–86.0)	100.0 (86.0–108.2)	103.4
c'	1.0 ± 0.1 (0.8–1.1)	1.8 ± 0.1 (1.7–2.0)	1.9 ± 0.2 (1.8–2.1)	1.3 ± 0.1 (1.2–1.4)	1.0 ± 0.05 (0.9–1.0)	0.9	0.9 ± 0.04 (0.9–1.0)	1.0 ± 0.7 (1.0–1.1)	1.0 (0.9–1.2)	1.0
V or T (%)	53.8 ± 1.5 (51.5–57.0)	–	–	–	53.7 ± 1.5 (52.0–57.5)	36.7	44.5 ± 0.5 (44.0–45.0)	–	44.0 (42.0–46.0)	–
Odontostyle	144.1 ± 4.2 (135.5–151.5)	81.6 ± 3.8 (77.5–86.5)	95.5 ± 3.5 (93.0–99.5)	116.7 ± 5.5 (111.0–122.0)	142.3 ± 6.6 (132.5–150.5)	133.5	148.5 ± 4.9 (142.9–156.5)	147.3 ± 1.2 (146.5–148.2)	151.9 (140.0–156.5)	132.3
Odontophore	89.4 ± 2.7 (85.0–95.0)	59.8 ± 2.0 (56.5–61.5)	65.5 ± 2.6 (64.0–68.5)	79.0 ± 2.6 (76.0–81.0)	85.9 ± 4.5 (80.5–93.0)	88.0	86.7 ± 2.3 (83.5–89.4)	89.4	86.4 (69.4–92.9)	74.7
Total stylet	233.5 ± 6.4 (223.5–245.0)	141.4 ± 4.9 (134.0–146.5)	161.0 ± 6.1 (157.0–168.0)	195.7 ± 7.8 (187.0–202.0)	228.2 ± 8.7 (213.5–241.0)	221.5	–	–	–	–
Replacement Odontostyle	–	100.7 ± 3.1 (95.5–104.0)	123.0 ± 0.9 (122.0–123.5)	143.0 ± 5.7 (136.5–147.0)	–	–	–	–	–	–
Lip width	15.4 ± 0.9 (14.0–17)	10.4 ± 0.2 (10.0–10.5)	11.8 ± 0.3 (11.5–12.0)	13.8 ± 0.4 (13.5–14.0)	15.9 ± 0.6 (14.5–16.5)	15.5	15.0 ± 0.3 (14.7–15.3)	15.0	14.0	13.5
Oa–guiding ring	128.0 ± 6.8 (116.0–139.0)	75.4 ± 2.4 (71.5–78.0)	88.0 ± 8.9 (78.0–95.0)	109.7 ± 15.5 (93.5–124.5)	117.9 ± 7.4 (108.5–129.5)	126.5	136.4 ± 6.1 (129.4–144.7)	135.0 ± 7.1 (130.0–140.0)	137.0 (117.1–145.9)	123.5
Tail length	45.3 ± 3.1 (40.5–50.0)	52.6 ± 1.8 (50.5–54.5)	58.2 ± 6.0 (51.5–63.0)	54.2 ± 2.5 (51.5–56.5)	41.8 ± 2.0 (38.5–44.5)	41.0	41.4 ± 2.3 (38.2–43.5)	50.0	42.6 (38.8–51.2)	43.5
J	16.0 ± 1.8 (13.0–19.5)	16.2 ± 2.9 (12.5–20.5)	17.3 ± 2.8 (15.5–20.5)	18.0 ± 1.3 (16.5–19.0)	17.3 ± 2.8 (13.5–23.5)	5.0	13.4 ± 0.8 (12.9–14.7)	14.4 ± 0.4 (14.1–14.7)	16.3 (14.1–18.2)	9.4
Spicules	–	–	–	–	–	74.0	–	84.0 ± 2.1 (82.3–85.2)	–	82.0
Lateral accessory piece	–	–	–	–	–	21.0	–	–	–	–
Suppl. 1-anus	–	–	–	–	–	24.5	–	–	–	–

Note: ^a^Measurements are in µm and in the form: mean  ±  standard deviation (range); ^b^a = body length/maximum body width; b = body length/pharyngeal length; c = body length/tail length; c’ = tail length/body width at anus; V = (distance from anterior end to vulva/body length)×100; T = (distance from cloacal aperture to anterior end of testis/body length)×100; J = hyaline tail region length.

### Description

#### Female

The description of female body of *Xiphinema histriae* is as follows: body is cylindrical with an open C-shaped when heat relaxed; cuticle is 3.1 (2.5–3.5) μm thick at mid-body; Lip region is flatly rounded, separated from body by a slight depression, 15.4 (14.0–17.0) μm wide and 8.0 (7.5–9.0) μm high; amphids are stirrup shaped and amphidial fovea aperture extending for *ca* 60.7 to 70.5% of lip region diam; odontostyle long and narrow, 1.6 times longer than odontophore; odontophore with well-developed flanges 15.6 (14.0–17.0) μm wide; pharynx extending to a terminal pharyngeal bulb with three nuclei: one dorsal gland nucleus (DN) located at the beginning of basal bulb (9.0–12.7%) and two ventro-sublateral nuclei (SVN) are located near to the middle of bulb (51.9–57.5%); glandularium is 152.8 (129.5–170.5) μm long; reproductive system didelphic-amphidelphic is with equally developed branches, and vulva slit-like and situated slightly posterior to mid-body; each branch comprises a reflexed ovary and a tubular oviduct with a developed *pars dilatata oviductus* separated from uterus by a sphincter; uteri tripartite with a long tubular part, consisting of a developed *pars dilatata uteri* link with a narrower, muscular tube-like portion containing crystalloid bodies distributed over the entire length, pseudo-Z-organ with weakly muscularized wall with numerous small dense granular bodies; ovejector is well developed, 22.2 (16.0–32.5) μm wide, and vagina is 30.4 (20.0–40.0) μm long or 47.4% (34.5–57.1%) of corresponding body width; prerectum is reaching around 8.9 to 11.5% of nematode body from the anus to anterior part; rectum is extending more or less than the body width at anus; and tail is short and hemispherical with a peg 6.0 to 9.5 μm long.

#### Male

Very rare, only one male specimen was found in both Spanish populations. It is morphologically similar to female except for the genital system. Male genital tract is diorchic with testes with multiple rows of spermatogonia. Spicules are moderately long, curved ventrally, and lateral guiding pieces 21.0 μm long. Tail is short and hemispherical with a peg 3.5 μm long. One pair is of adanal supplements and seven of mid-ventral supplements.

#### Juveniles

Three juvenile stages (J2, J3, and J4) were found and they were basically similar to adults, except for their smaller size, shorter tails, and sexual characteristics (Figs. 1, 2). The tails of juvenile stages become progressively wider after each moult. All of the stages are distinguishable by relative body lengths, functional, and replacement odontostyle (Robbins et al., 1996).

##### Locality and habitat

Spanish populations of *Xiphinema histriae* were collected in the rhizosphere of Portuguese oak (*Quercus faginea* Lam.) and black pine (*Pinus nigra* Arnold) at Arroyo Frío and Nava de San Pedro, Cazorla, Jaén Province, Spain.

##### Remarks

The two amphimictic populations of *X. histriae* agree fairly with studied paratypes (Fig. 3) and original description of *X. histriae* by Lamberti et al. (1993a, 1993b). According to the polytomous key (Loof and Luc, 1990), these populations belong to the *X.* non-*americanum* Group 5 and has the following specific *α*-numeric codes: A4, B23, C5a, D56, E6, F45, G3, H2, I3, J5, K?, L1, which fits with the original description of *X. histriae*, except in having bigger values of V (51.5–57.0 vs 44.0–44.5), shorter oral aperture-guiding ring length (116.0–139.0 μm vs 129.4–144.7 μm), and spicule length (74.0 μm vs 82.3–85.2 μm). No juvenile stages were described in the original description. This is the first time that J2 to J4 juvenile stages were detected and described, being similar to adults, except in body length, tail morphology, and sexual characteristics. Additionally, females of the Spanish populations of *X. histriae*, a pseudo-Z-organ with weakly muscularized wall, containing numerous small dense granular bodies was observed, which differ from the original description by Lamberti et al. (1993a, 1993b). This pseudo-Z-organ was also confirmed in detailed examination of paratypes (Fig. 3). Therefore, *X. histriae* should be placed in morphospecies Group 5. To our knowledge, this is the first report of this species in Spain.

**Figure 4:**
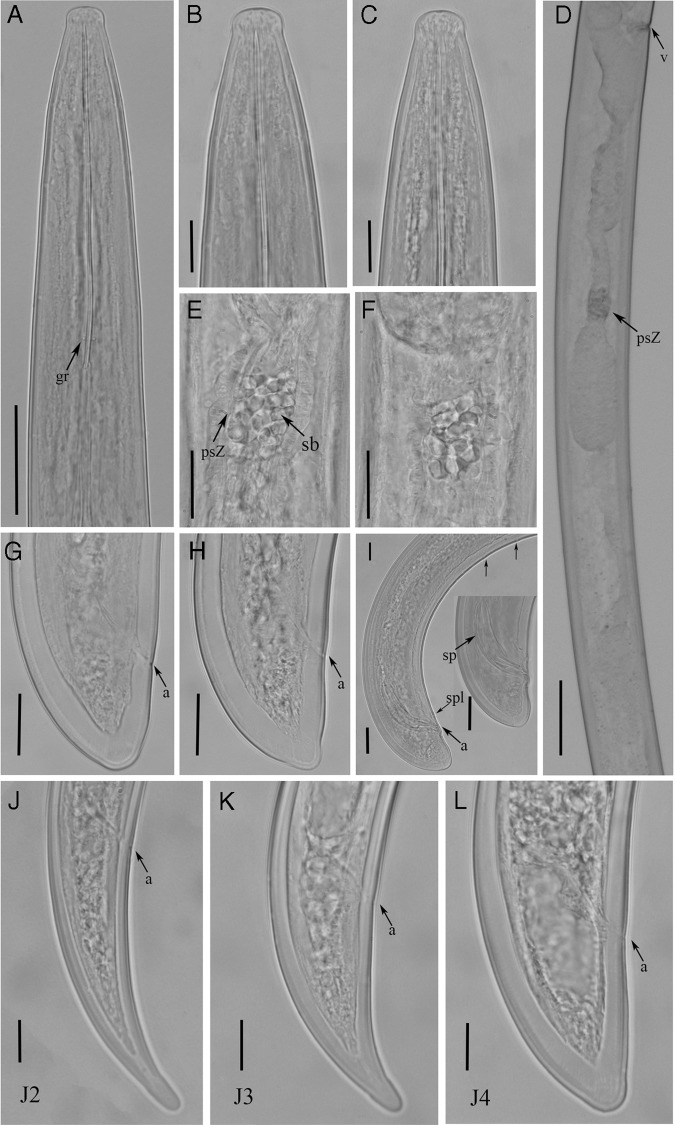
Light micrographs of *Xiphinema lapidosum* (Roca and Bravo, 1993). Females: A, Pharynx; B to C, Lip regions; D, Gonad; E, F, Details of pseudo-Z-organ; G, H, Tail regions; Males: I, Tail region of male; Juveniles: J to L, Tail region of 2nd, 3rd and 4th stage juveniles. Abbreviations: a, anus; gr, guiding ring; psZ, pseudo-Z-organ; sb, sclerotized bodies; sp, spicule; spl, supplements; v, vulva. (Scale bars: A = 40 μm; B, C  = 15 μm; D = 65 μm; E–I = 20 μm; J–L = 10 μm.)

**Figure 5:**
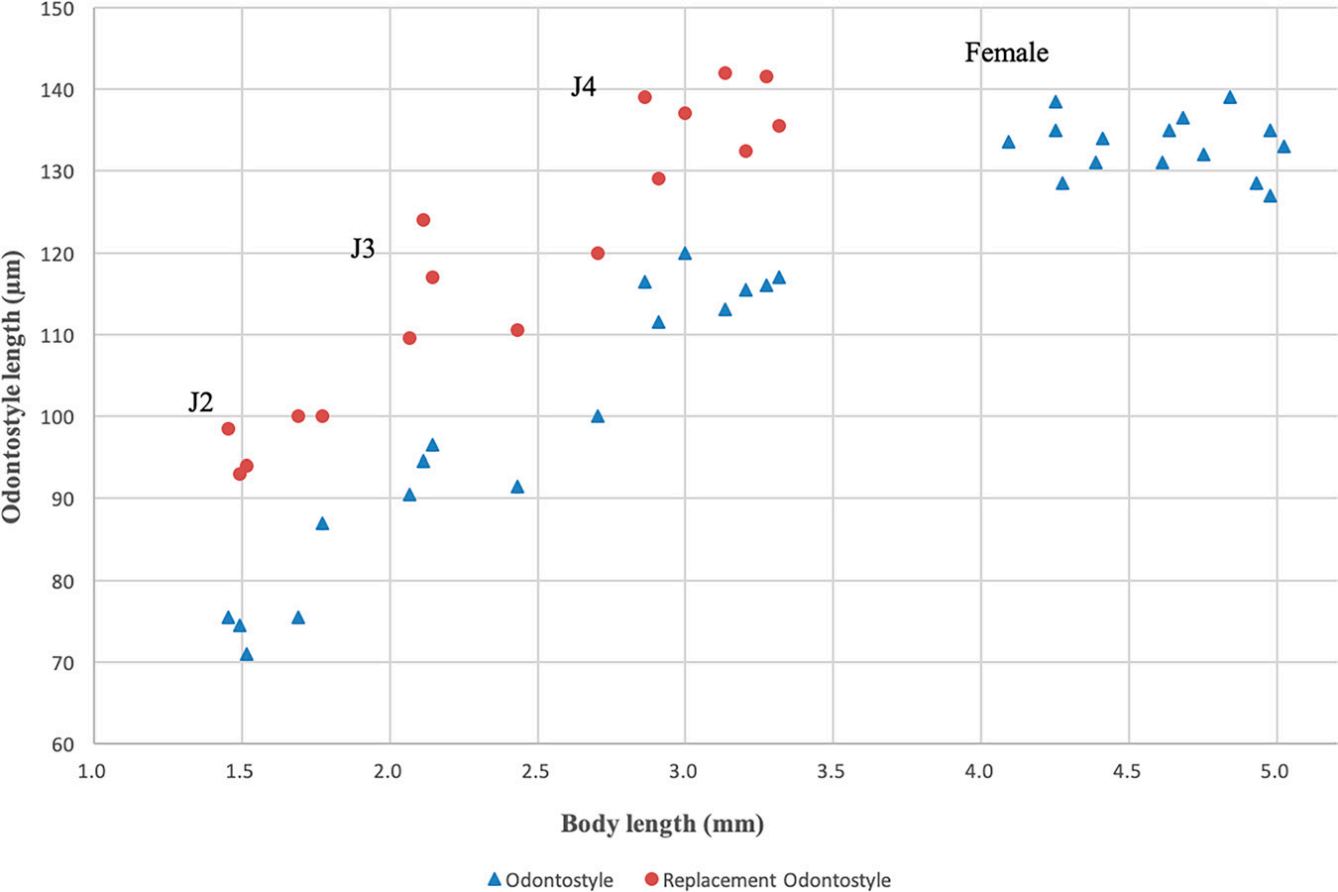
Relationship of body length to length of functional and replacement odontostyle (▴= Odontostyle and •=  Replacement odontostyle); length in three developmental stages and mature females of *Xiphinema lapidosum*.

**Figure 6:**
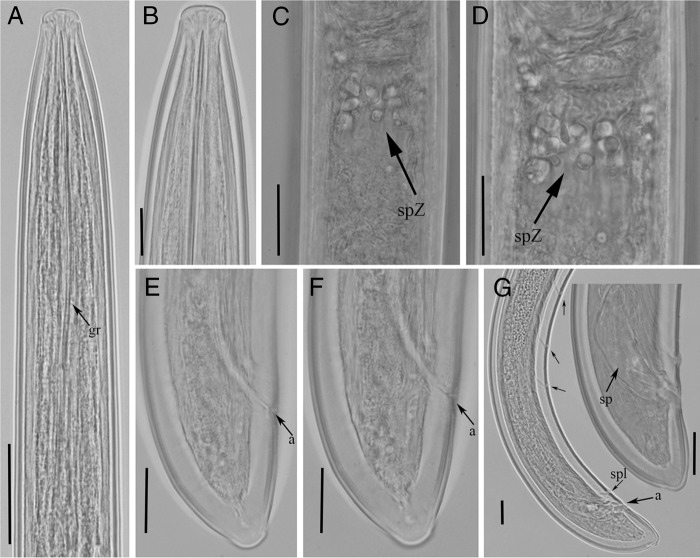
Light micrographs of *Xiphinema lapidosum* paratypes (Roca and Bravo, 1993). Female: A, Pharynx; B, Lip region; C, D, Details of pseudo-Z-organ; E, F, Tail regions; Males: G, Tail region of male. Abbreviations: a, anus; gr, guiding ring; psZ, pseudo-Z-organ; sp, spicule; spl, supplements. (Scale bars: A = 40 μm; B = 15 μm; C-G = 20 μm.)

*Xiphinema lapidosum* Roca and Bravo (1993).

(Figs. 4–6; Table 3).

**Table 3.** Morphometrics of *Xiphinema lapidosum* (Roca and Bravo, 1993) from culti**v**ated olive at Aroche (Huelva, Spain). All measurements are in µm and in the form: mean  ±  s.d. (range)^a^.

						Roca and Bravo (1993) Quinta do Rogelo, Silves, Faro, Portugal/broad-beans and peas	
Characters/ratios	Females	Males	J2	J3	J4	Females	Males
n	10	5	2	5	7	14	7
L^b^	4,600 ± 271.5 (4,250–5,023)	4,618 ± 415.4 (4,091–4,977)	(1,689–1,773)	2,292 ± 270.7 (2,068–2,704)	3,101 ± 178.9 (2,864–3,318)	4,300 ± 344 (3,700–4,600)	4,300 ± 310 (3,900–4,800)
a	71.5 ± 4.9 (64.9–75.5)	70.9 ± 6.7 (63.4–79.6)	(57.3–59.1)	63.4 ± 3.5 (59.6–68.5)	67.0 ± 6.3 (59.3–77.6)	80.0 ± 5.3 (70.4–88.2)	85.0 ± 6.5 (75.5–95.5)
b	9.8 ± 0.6 (8.8–10.9)	10.9 ± 1.5 (9.1–12.3)	4.5	6.9 ± 0.9 (6.1–7.8)	7.4 ± 0.6 (6.4–8.1)	9.5 ± 0.8 (7.8–10.7)	9.0 ± 0.85 (7.5–10.0)
c	113.2 ± 8.2 (98.8–123.6)	118.4 ± 9 (109–129.8)	(23.2–24.0)	40.5 ± 5.5 (35.3–49.2)	67.7 ± 4.3 (62.9–74.5)	110.0 ± 12.16 (92.0–132.0)	113.2 ± 9.73 (101.5–130.0)
c'	0.9 ± 0.07 (0.8–1.0)	0.9 ± 0.08 (0.8–1.0)	(3.1–3.4)	2.2 ± 0.2 (2.0–2.5)	1.3 ± 0.1 (1.2–1.5)	1.04 ± 0.07 (0.92–1.13)	0.99 ± 0.07 (0.92–1.09)
V or T (%)	46.0 ± 2.8 (40.6–51.0)	50.3 ± 5.6 (45.2–56.3)	–	–	–	43.4 ± 1.28 (41.0–46.0)	–
Odontostyle	133.0 ± 2.5 (128.5–136.5)	133.3 ± 5.5 (127.0–139.0)	(75.5–87.0)	94.6 ± 3.8 (90.5–100.0)	115.6 ± 2.8 (111.5–120.0)	134.5 ± 3.71 (124.5–139.5)	136.0 ± 3.88 (130.5–142.5)
Replacement odontostyle	–	–	100.0	116.1 ± 5.5 (109.5–124.0)	136.6 ± 4.7 (129.0–142.0)	–	–
Odontophore	82.4 ± 4.6 (72.0–88.5)	78.0 ± 3.9 (73.0–83.5)	(53.0–55.5)	58.2 ± 3.5 (54.0–63.0)	70.9 ± 3.1 (66.5–76.0)	71.5 ± 1.39 (69.0–73.5)	71.5 ± 2.67 (67.5–74.5)
Lip region width	14.6 ± 0.5 (14.0–15.5)	14.7 ± 0.8 (13.5–15.5)	10.0	11.5 ± 0.6 (10.5–12.0)	12.6 ± 1.3 (10.5–14.0)	15.0 ± 0.60 (14.0–16.0)	15.0 ± 0.31 (14.5–15.5)
Oral aperture-guiding ring	122.5 ± 6.9 (111.5–135.0)	117.9 ± 3.0 (113.0–120.5)	(65.0–73.0)	81.5 ± 4.2 (76.5–86.0)	98.2 ± 7.3 (84.0–106.0)	116.5 ± 7.03 (97.5–127.5)	121.5 ± 5.94 (115.5–130.5)
Tail length	40.8 ± 2.8 (36.5–44.0)	39.0 ± 2.0 (36.5–42.0)	(70.5–76.5)	57.0 ± 3.5 (51.5–59.5)	46.5 ± 3.1 (43.0–52.0)	39.0 ± 2.04 (34.0–41.0)	38.5 ± 2.77 (35.4–43.0)
Hyaline tail	11.2 ± 1.5 (9.5–14.0)	9.3 ± 1.4 (7.5–10.5)	14.5	13.7 ± 3.4 (9.0–18.5)	13.3 ± 0.8 (12.0–14.0)	11.5 ± 1.8 (9.0–15.5)	11.0 ± 1.59 (8.5–13.0)
Spicules	–	66.2 ± 3.1 (63.0–71.0)	–	–	–	–	64.5 ± 3.02 (60.0–68.0)
Lateral accessory piece	–	18.0 ± 1.5 (16.5–19.5)	–	–	–	–	15.5 ± 1.55 (13.5–18.0)

Note: ^a^Measurements are in µm and in the form: mean ± standard deviation (range); ^b^a = body length/maximum body width; b = body length/pharyngeal length; c = body length/tail length; c’ = tail length/body width at anus; V = (distance from anterior end to vulva/body length)×100; T = (distance from cloacal aperture to anterior end of testis/body length)×100; J = hyaline tail region length.

### Description

#### Female

The female body of *Xiphinema lapidosum* is as follows: body is cylindrical, slightly tapering anteriorly and posteriorly and assuming a hook-shape upon fixation; cuticle appearing smooth, 5.0 (3.5–6.5) μm thick at the middle body; lip region is flatly rounded, separated by a weak depression; odontostyle is robust, and odontophore is with well-developed basal flanges (10.5–14 μm wide); guiding ring is double; pharynx is extending to a terminal pharyngeal bulb with three nuclei with one dorsal gland nucleus located at the beginning of pharyngeal bulb (DN = 8.5–10.5%), while two subventrolateral nuclei located at middle of bulb (SN12 = 56–60%); pharyngeal basal bulb 127 to 148 μm long and 24.5 to 35 μm diam; glandularium is 111.5 (106.5–115) μm long; female reproductive system is didelphic, with two complete genital branches equally developed, each 541 (465–580) μm long; the length of ovaries is variable, and a *pars dilatata oviductus s*eparated from the uterus by a conspicuous sphincter muscle, tripartite uterus consisting of a *pars dilatata uteri* followed by a tubular portion, a pseudo-Z-organ, a dilated part and an ovejector; pseudo-Z-organ well developed with a thick wall and longitudinal folding is easy to observe, comprising 15 to 20 sclerotized bodies of large size, but all of them of variable size; no spines or different structures are observed in the uterus; vulva is a transverse slit, vagina 33.0 (30.5–37.5) μm wide and perpendicular to body-axis, ovejector well developed, 50.5 (40.5–58.5) μm wide, extending inwards more than half of corresponding body diam; and tail short, convex dorsally and ending with bulge.

#### Male

Males are common but less frequent (50%) than female. They are morphologically similar to female except for the genital system; spicules are curved, lateral guiding pieces well sclerotized; tail is conoid with one pair of adanal supplements and five mid-ventral supplements (Table 3, Fig. 4).

#### Juveniles

Three juvenile stages (J2, J3, and J4) were found and they were basically similar to adults, except for their smaller size, shorter tails, and sexual characteristics (Table 3, Fig. 4). Tail becomes progressively wider and shorter after each moult.

##### Locality and habitat

The population was collected from the rhizosphere of cultivated olive (*Olea europaea* subsp. *europaea* L.) at Aroche, Huelva province, Spain.

##### Remarks

The amphimictic population of *X. lapidosum* from Aroche (Huelva province) corresponds fairly well with the original description (Roca and Bravo, 1993) and the studied paratypes from USDA (Fig. 6). Observations on the general morphology indicate that this *Xiphinema* population belongs to the *X.* non-*americanum* morphospecies Group 5 (Loof and Luc, 1990), and has the following specific *α*-numeric codes: A4, B2, C5b, D6, E456, F45, G3, H2, I3, J3, K?, L2. In addition, female and male morphometrics fit with those provided in the original description, except in having slightly longer values of body length (4,250–5,023 μm vs 3,700–4,600 μm), odontophore length (72.0-88.5 μm vs 69.0–73.5 μm), and slightly smaller values of c’ (0.83–1.04 vs 0.92–1.13). Since juveniles were not described in the original description, the J2 to J4 juvenile stages of Aroche population were described herein for the first time. To our knowledge, this is the first report of this species for Spain.

### Phylogenetic relationships of *Xiphinema histriae* and *Xiphinema lapidosum*

Amplification of D2 to D3 expansion segments of 28S rRNA, ITS1 and the partial CoxI gene from *X. histriae* and *X. lapidosum* yielded a single fragment of *ca* 900, 1,100, and 500 bp, respectively. Six new D2 to D3 of 28S rRNA, three ITS1, and five partial CoxI gene sequences were obtained in the present study. *Xiphinema histriae* showed a high molecular similarity for D2 to D3 expansion segments of 28S rDNA, only one variable position was found between the sequences obtained from three female specimens (MK801302-MK801305). These sequences matched well with *X.* non*-americanum* group species deposited in GenBank and showed 97% similarity (differing from 21 to 24 nucleotides and from 1 to 4 indels) with *X. hispidum* (Roca and Bravo, 1994), *X. hispanum* (Lamberti et al., 1992), *X. adenohystherum*, and *Xiphinema celtiense* (Archidona-Yuste et al., 2016a, 2016b, 2016c). ITS1 region from *X. histriae* (MK801298-MK801299) also showed similarity with *X. hispanum*, *X. adenohystherum*, and *X. hispidum* displaying similarity values of 88, 86, and 84% (differing from 129 to 152 nucleotides and from 31 to 43 indels), respectively. In addition, the two new partial CoxI sequences of *X. histriae* (MK796911-MK796912) showed similarity values from 86% to 77% with all *X.* non*-americanum* group species in GenBank (differing from 49 to 80 nucleotides). Non intra-specific variation for this region was found among two studied individuals.

The closet species regarding D2 to D3 segments of *X. lapidosum* (MK801306-MK801307) were *X. lupini* (Roca and Pereira, 1993), 97% similar (differing from 22 to 24 nucleotides and from 3 to 4 indels), and *X. turcicum*, 88% similar (similarity of 83 nucleotides and 22 indels). Similarly, *X. lupini* was the most related species for the ITS1 rRNA region showing a similarity value of 87% with *X. lapidosum* (MK801300). Scarce similarity was found with the rest of *Xiphinema* spp. deposited in GenBank, showing coverage values below 30% with all of them. Finally, three new CoxI from *X. lapidosum* (MK796913-MK796915) were obtained in this study, being clearly different to the other accession from *X.* non-*americanum* group species deposited in GenBank and showing similarity values from 82 to 73% with all of them, being *X. lupini* the closet species (82% similar, 66 nucleotides and no indels) as in the D2 to D3 and ITS1 regions. No intra-specific variation was found between D2 and D3 and CoxI sequences from *X. lapidosum* obtained in this study (MK801306-MK801307, MK796913-MK796915).

**Figure 7:**
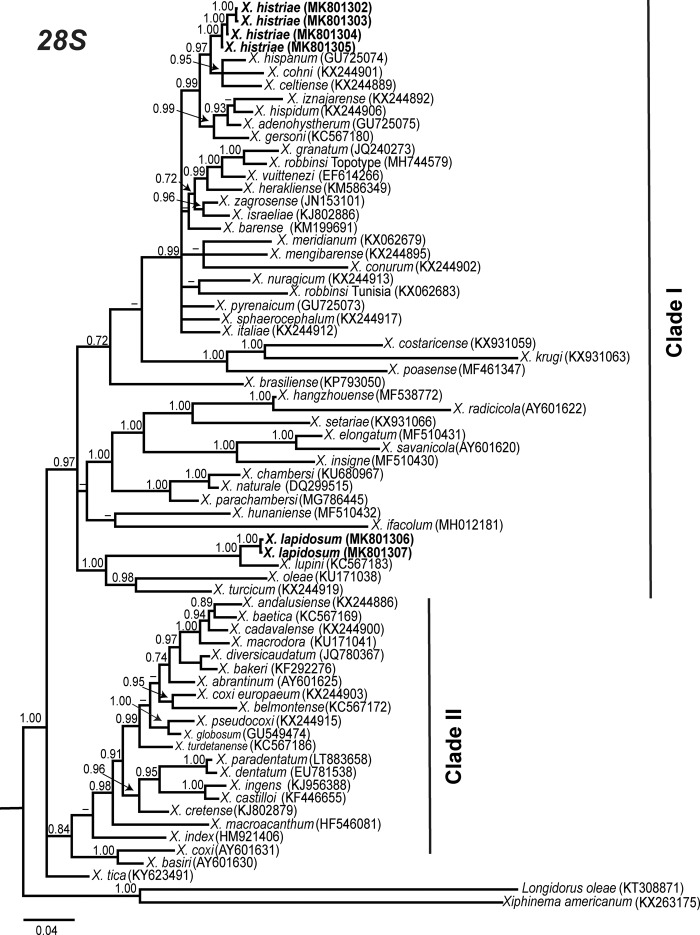
Phylogenetic relationships within the *Xiphinema* non*-americanum* group complex. Bayesian 50% majority rule consensus tree as inferred from D2 to D3 expansion segments of 28S rRNA sequence alignment under the general time-reversible model of sequence evolution with correction for invariable sites and a gamma-shaped distribution (GTR + I + G) (lnL = 11,543.7822; AIC = 23,383.5644; freq A = 0.2502; freq C = 0.2298; freq G = 0.2995; freq T = 0.2205; R(a) = 0.9908; R(b) = 2.7656; R(c) = 2.4778; R(d) = 0.4894; R(e) = 4.2554; R(f) = 1.0000). Posterior probabilities greater than 0.70 are given for appropriate clades. Newly obtained sequences in this study are shown in bold. Scale bar = expected changes per site.

**Figure 8:**
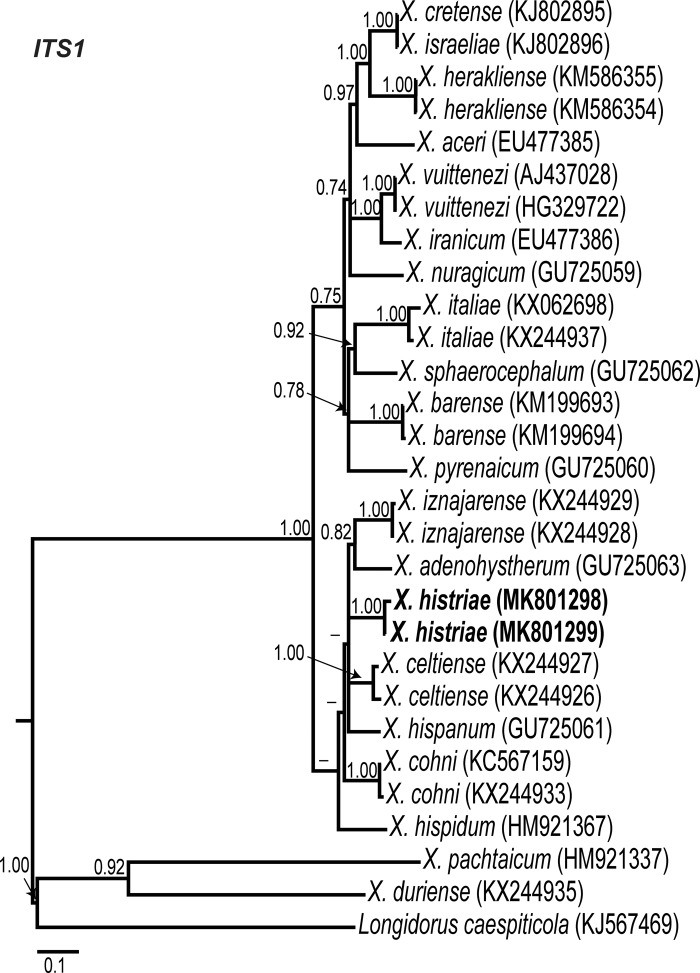
Phylogenetic relationships within the *Xiphinema* non-*americanum* group complex. Bayesian 50% majority rule consensus tree as inferred from ITS1 rRNA gene sequence alignment under the general time-reversible model of sequence evolution with correction for invariable sites and a gamma-shaped distribution (GTR + I + G) (lnL = 6,024.1284; AIC = 12,068.2568; freq A = 0.2316; freq C = 0.2224; freq G = 0.3009; freq T = 0.2451; R(a) = 0.7019; R(b) = 4.6043; R(c) = 2.0272; R(d) = 0.6248; R(e) = 7.4428; R(f) = 1.0000). Posterior probabilities greater than 0.70 are given for appropriate clades. Newly obtained sequences in this study are shown in bold. Scale bar = expected changes per site.

**Figure 9:**
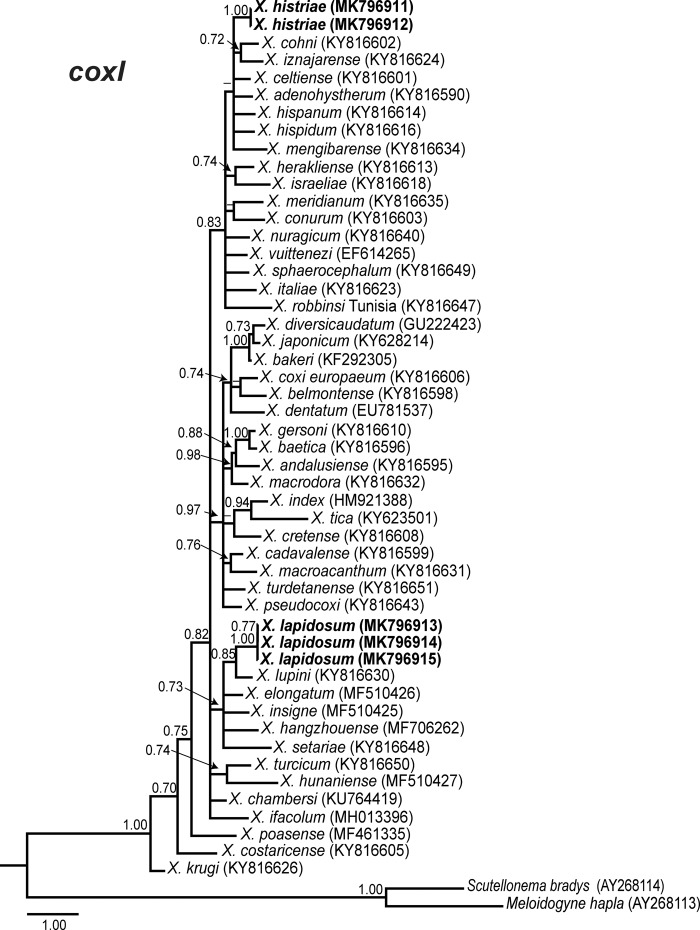
Phylogenetic relationships within the *Xiphinema* non-*americanum* group complex. Bayesian 50% majority rule consensus tree as inferred from partial cytochrome c oxidase subunit I (CoxI) gene sequence alignment under the general time-reversible model of sequence evolution with correction for invariable sites and a gamma-shaped distribution (GTR + I + G), (lnL = 8,561.5874; AIC = 17,347.1747; freq A = 0.3687; freq C = 0.1301; freq G = 0.1382; freq T = 0.3630; R(a) = 3.3231; R(b) = 22.8405; R(c) = 2.0144; R(d) = 10.5400; R(e) = 75.4228; R(f) = 1.0000). Posterior probabilities greater than 0.70 are given for appropriate clades. Newly obtained sequences in this study are shown in bold. Scale bar = expected changes per site.

Phylogenetic relationships among *Xiphinema* non-*americanum* group species inferred from analyses of D2 to D3 expansion segments of 28S, ITS1, and the partial CoxI gene sequences using BI are given in Figures 7 to 9, respectively. Poorly supported clusters were not explicitly labelled. The 50% majority rule consensus 28S rRNA gene BI tree of *X.* non-*americanum* group spp. based in a multiple edited alignment including 70 sequences and 771 total characters showed two clearly separated (PP = 1.00) major clades (Fig. 7). Clade I grouped species from all morphospecies groups, including the new accessions obtained in this study of *X. histriae* and *X. lapidosum*. Clade II was not well supported (PP = 0.84) and was mostly composed by species from the morphospecies Group 5, except for *X. tica*, *X. bakeri*, and *X. index* which belong to Groups 4, 7, and 8, respectively. *Xiphinema histriae* (MK801302-MK801305) occupies a superior position within this major clade I clustering with *X. hispanum*, *X. celtiense*, and *X. cohni* in a well-supported subclade (PP = 0.97). On the contrary, *X. lapidosum* (MK801306-MK801307) occupied a basal position and seemed to be related with *X. lupini*, *X. turcicum*, and *X. oleae* since all of them formed a well-supported subclade (PP = 0.99). The low similarity and small coverage between the ITS1 region from *X. lapidosum* and the rest of the ITS1 sequences available in GenBank made it impossible to perform a phylogenetic analysis for this region. For *X. histriae*, only ITS1-related sequences were used, the edited alignment generated for the 29 sequences of ITS1 was of 1,104 characters after discarding ambiguously aligned regions. This ITS phylogenetic tree (Fig. 8) showed two major clades (PP = 1.00), similar to those obtained for D2 to D3 region. *Xiphinema histriae* (MK801298-MK801299) appeared in the basal major clade but their phylogenetic position was not well resolved for this marker (Fig. 8). The CoxI region using a multiple alignment of 52 sequences and 390 characters after editing was used to obtain the 50% majority rule BI tree (Fig. 9). The position of *X. histriae* (MK796911-MK796912) was not well-defined, but clustering with *X. hispanum*, *X. hispidum*, *X. cohni*, and *X. celtiense*. By contrast, the relationship among *X. lapidosum* (MK796913-MK796915) and *X. lupini* was maintained.

## Discussion

This study aimed to provide and to characterize morphometrically and molecularly two *Xiphinema* species belonging to *Xiphinema* non-*americanum* Group 5 from Spain, and to carry out an updated phylogenetic study of both species within the *X.* non-*americanum* group species. To date, this is the first record of the occurrence of *X. histriae* and *X. lapidosum* in Spain and the first time that describes the molecular characterization and the juvenile stages of both species.

*Xiphinema histriae* was originally described from Italy associated with grapevine (Lamberti et al., 1993a, 1993b, and later on, reported from the rhizosphere of wild growing grape (*Vitis vinifera* ssp. *silvestris*) in Austria (Tiefenbrunner and Tiefenbrunner, 2004). Based on the detailed study of paratypes and both Spanish populations described here, we detected that this species is characterized by having a pseudo-Z-organ with weakly muscularized wall with numerous small dense granular bodies against that initially described by Lamberti et al. (1993a, 1993b). Therefore, *X. histriae* must be transferred to morphospecies Group 5 (Loof and Luc, 1990). This study illustrates the importance of paratypes deposited in different official collections and reference nematology laboratories of nematodes, which are provided as a useful tool in the accurate identification and revision of nematodes species. On the other hand, *X. lapidosum* was, first, described from the rhizosphere of broad bean and pea in the south of Portugal (Roca and Bravo, 1993) and now it is reported from cultivated olive at Huelva, southwestern Spain. These data suggest that *X. histriae* may have a wider distribution than that described until now (including agricultural and natural ecosystems), and *X. lapidosum* may be an Iberian endemism, also associated with cultivated hosts.

The use of different ribosomal and mitochondrial markers in this study, D2 to D3, ITS1, and partial CoxI, provides a precise and unequivocal tool for the identification of *X*. *histriae* and *X. lapidosum.* Phylogenetic analyses based on D2 to D3, ITS1, and CoxI gene using BI resulted in a consistent position for *X. histriae* and *X. lapidosum. Xiphinema histriae* clustered with *Xiphinema* species from morphospecies Group 5, such as *X. hispanum*, *X. cohni*, *X. celtiense*, and *X. hispidum*, while *X. lapidosum* seems to be related with *X. lupini* because of both species clustered together in all the analyses carried out in this study. The present study on the phylogeny based on D2 to D3 segments supported a very weak correlation in the phylogenetic relationships among the different morphospecies groups within *Xiphinema*, a finding already reported by several authors namely, Gutiérrez-Gutiérrez et al., 2013; De Luca et al., 2014; Tzortzakakis et al., 2014, 2015; Archidona-Yuste et al., 2016a, 2016b, 2016c).

In summary, this study highlighted the diagnosis of *Xiphinema* non-*americanum* group species because a large number of species and the lack of good diagnostic characteristics among the *X.* non*-americanum* group (Loof and Luc, 1990; Loof et al., 1996). For this reason, we recommend the use of integrative taxonomy that are crucial for accurately identify species and better understanding of the present geographical distribution and host range of *X.* non*-americanum* group species. In this case, we provide new morphological and molecular data for the precise identification of these species, the first reports of these species in Spain, new hosts, and their phylogenetic position in the *Xiphinema* genus.
